# Criticality predicts maximum irregularity in recurrent networks of excitatory nodes

**DOI:** 10.1371/journal.pone.0182501

**Published:** 2017-08-17

**Authors:** Yahya Karimipanah, Zhengyu Ma, Ralf Wessel

**Affiliations:** Department of Physics, Washington University in St. Louis, St. Louis, MO, United States of America; McGill University Department of Physiology, CANADA

## Abstract

A rigorous understanding of brain dynamics and function requires a conceptual bridge between multiple levels of organization, including neural spiking and network-level population activity. Mounting evidence suggests that neural networks of cerebral cortex operate at a critical regime, which is defined as a transition point between two phases of short lasting and chaotic activity. However, despite the fact that criticality brings about certain functional advantages for information processing, its supporting evidence is still far from conclusive, as it has been mostly based on power law scaling of size and durations of cascades of activity. Moreover, to what degree such hypothesis could explain some fundamental features of neural activity is still largely unknown. One of the most prevalent features of cortical activity in vivo is known to be spike irregularity of spike trains, which is measured in terms of the coefficient of variation (CV) larger than one. Here, using a minimal computational model of excitatory nodes, we show that irregular spiking (*CV* > 1) naturally emerges in a recurrent network operating at criticality. More importantly, we show that even at the presence of other sources of spike irregularity, being at criticality maximizes the mean coefficient of variation of neurons, thereby maximizing their spike irregularity. Furthermore, we also show that such a maximized irregularity results in maximum correlation between neuronal firing rates and their corresponding spike irregularity (measured in terms of CV). On the one hand, using a model in the universality class of directed percolation, we propose new hallmarks of criticality at single-unit level, which could be applicable to any network of excitable nodes. On the other hand, given the controversy of the neural criticality hypothesis, we discuss the limitation of this approach to neural systems and to what degree they support the criticality hypothesis in real neural networks. Finally, we discuss the limitations of applying our results to real networks and to what degree they support the criticality hypothesis.

## Introduction

Having fundamental principles underlying neural dynamics and function entails a unifying theory that captures various universal features of cortical activity at different levels. One of the most prevalent features of single-neuron spiking in cerebral cortex is irregular spiking [1], which is characterized by mean *coefficient of variation* (CV) being larger than one. On the other hand, collective behavior of neural systems is characterized by complex spatiotemporal patterns of activity, including scale-free activity [2–10]. This scale invariance is manifested by the power law distributed cascades of activity, or the so-called *neuronal avalanches*, and is predicted to occur for a network at a critical state; a state of balanced propagation at the transition between two phases of short lasting and chaotic activity [11–13]. These observations at two adjacent levels of brain organization raise the question to what extent the network state controls the variability of single-neuron spiking?

Theoretical studies have linked the prevalence of irregular spiking to the fluctuations of synaptic inputs at the sub-threshold regime [14, 15]. It has also been shown that balanced networks of leaky integrate-fire neurons may naturally generate irregular spiking (*CV* > 1) when the mean synaptic strength goes above a critical value [16]. However, none of those scenarios has been directly linked to the scale free nature of cortical activity. On the other hand, the prevalence of scale free network activity with certain critical exponents supports the hypothesis that cortical networks operate at a directed percolation (DP) phase transition [17]. These two findings beg the question whether these two separate universal features, irregular spiking of single neurons and scale free population activity, both can possibly be explained based on a unified theory.

To address this question, using a computational model we provide an alternative scenario, by which the onset of irregular spiking can simply emerge at a DP critical transition between two phases of short lasting activity and chaos. Further, we show that criticality could enhance the spike irregularity even at the presence of other mechanisms for irregular spiking (*CV* > 1), thereby leading to maximized CV. Moreover, we hypothesize that such maximum irregularity occurs as a result of the onset of (large) recurrent activity near criticality. Thereafter, using our model we verify our hypothesis by showing that neurons with higher in-degree connections tend to show higher irregularity. As a result, in addition to spike irregularity, we show that criticality also predicts maximum (positive) correlation between a number of single-neuron properties such as their CV’s and firing rates. Our findings provide us with robust measures of critical dynamics, which not only could further our understanding about the implications of criticality on network dynamics, but at the same time could be used as hallmarks of criticality at the single-unit level. Finally, we discuss the limitations of this picture when it comes to the presence of inhibition, arguing that our model predictions could break down in the regime of synchronized irregular (SI) activity, when the inhibition is suppressed and the network shows synchronization and irregular spiking simultaneously [18–20]. However, the question whether there exists a critical point/line along which real neural networks could show a DP phase transition is still unknown.

## Materials and methods

### Binary probabilistic network model

We simulated a model network consisting of excitatory binary probabilistic model neurons with sparse connectivity and external inputs. The model has been studied both numerically and analytically [21–23], and is in fact the most natural extension of a branching process to a recurrent network. Network size ranged from 5000 to 20000 model neurons. The strength of the connection from neuron *j* to neuron *i* is quantified in terms of the transition probability *P*_*ij*_, which is the probability that a spike in neuron *j* causes a spike in neuron *i* in the next simulation time step. For a network of *N* neurons and an average connectivity *K*, each neuron is connected to *N* − 1 other neurons with probability *K*/*N*. For each (on average) *K*(*N* − 1) connections among neurons a *P*_*ij*_ is assigned by drawing a random number from uniform distribution in the interval $\left[0\;\;\frac{2}{K}\right]$. With a sufficiently large *N*, this yields a network with a normally distributed connectivity with average *K* and a transition matrix *P*_*ij*_ with maximum (absolute value) eigenvalue of 1. In order to push the network toward the sub-critical (super-critical) regime, we can simply multiply all *P*_*ij*_’s by a factor smaller(greater) than one. At each time step, the state of all neurons are updated synchronously according to the following probability:

$$\begin{array}{c} P_{i}\left(t+1\right)\equiv P\left(i_{t+1}|J\left(t\right)\right)=1-(1-\eta_{i}\left(t\right))\prod_{j\in J(t)}\left(1-P_{ij}\right) \end{array}$$ (1)

where *P*(*i*_*t* + 1_|*J*(*t*)) denotes the probability of neuron *i* spiking at time *t* + 1 given that the set of neurons *j* ∈ *J*(*t*) spike at the previous time-step *t*, where *η*_*i*_(*t*) is the probability of neuron *i* spiking only due to external input, and *J*(*t*) denotes the set of all neurons that spike at time *t*. It should be noted that this equation is valid when the spikes of all neurons in *j* ∈ *J*(*t*) can be assumed as independent with a good approximation. In other words, it entails a locally tree-like propagation of activity, which has been shown to be a good approximation for a wide range of connectivities [22]. At the large size limit (*N* ≫ 1) and assuming that *P*_*ij*_ inversely scales with *N* ($P_{ij}∼\frac{1}{K}$) the above equation can be approximated as follows:

$$\begin{array}{c} P_{i}\left(t+1\right)=1-(1-\eta_{i}\left(t\right)-(1-\eta_{i}\left(t\right))\sum_{j\in J(t)}P_{ij})+O\left(P_{ij}^{2}\right) \end{array}$$ (2)

Ignoring the higher order terms in Eq (2) leads to obtaining an update rule as follows:

$$\begin{array}{c} X_{i}\left(t+1\right)=\Theta [(1-\eta_{i}\left(t\right))\sum_{j}P_{ij}X_{j}\left(t\right)+\eta_{i}\left(t\right)-\xi_{i}\left(t\right)] \end{array}$$ (3)

where the binary state *X*_*i*_(*t*) of neuron *i* denotes whether the model neuron spikes (*X*_*i*_(*t*) = 1) or does not spike (*X*_*i*_(*t*) = 0) at time *t*. Here, *ξ*(*t*) is a random number in [0 1] drawn from a uniform distribution, and Θ is the step function. In addition to the update rule (3), a refractory period of two time-steps was implemented. The external input *η*_*i*_(*t*) was chosen to be smaller than the transition probability *P*_*ij*_, which itself is small for large networks, *P*_*ij*_ ∼ 1/*K*. The maximum eigenvalue λ of the transition probability matrix *P*_*ij*_ describes the network state at the infinite-size limit: λ ≈ 1 denotes the critical regime, whereas λ < 1 (λ > 1) denotes subcritical (supercritical) regime. However, for finite-sized networks we evaluate the critical point based on the peak of average population coupling (see below). The *P*_*ij*_ values were drawn from a uniform distribution and rescaled afterwards to reach the desired maximum eigenvalue λ.

The external input was simulated in three different ways. For random external drive, *η*_*i*_(*t*) can be modeled either as a constant or to better emulate natural stimuli, it can be modeled as random asynchronous stimuli. The asynchronous input was simulated as a binary Poisson process followed by smoothing with a Gaussian filter with an arbitrary width of 20 time steps and multiplied by an amplitude factor *η*_0_ between 0 and 1. The reason for using a Gaussian filter is to give the external drive a smooth temporal structure, making it more naturalistic. The synchronous input was simulated with replicating a single binary Poisson process smoothed with a Gaussian filter with a width of 100 time-steps. In order to impose some variability among the stimuli received by different neurons each smoothed Poisson process was multiplied by a (arbitrary) factor of *η*_0_ + *ϵζ*(*t*) where *η*_0_ = *ϵ* = 0.2 and *ζ*(*t*) was drawn from a normal distribution.

### Statistical measures

We analyzed the simulated spike trains with respect to different statistical measures: neuronal avalanches, coefficient of variation, population coupling, and change in the mean response. Following commonly-used procedures [2], a neuronal avalanche was defined as an episode of continuous (each time step) network spiking, framed by time steps of no spikes. Avalanche size was taken as the number of spiking neurons during each avalanche. However, it should be noted that the avalanche analysis in this study is mostly for the sake of illustration, as the network model has been already studied rigorously and is known to exhibit power law distributed avalanches at the critical point of λ = 1 [21, 22].

The single-neuron spike train variability was quantified using the coefficient of variation *CV* ≡ *σ*_*isi*_/*μ*_*isi*_, defined as the ratio of the standard deviation *σ*_*isi*_ and the mean *μ*_*isi*_ of the inter-spike-interval (*ISI*) distribution for a given neuron. We managed simulation times to be sufficiently long to ensure stable *CV* values.

The coordination within the network was quantified using the population coupling, which is defined as the zero-lag cross-correlation between the spike train *X*_*i*_(*t*) of neuron *i* and the remaining network activity *N*_*i*_(*t*) = ∑_*j* ≠ *i*_ *X*_*j*_(*t*) from all other spike trains. The cross-correlation could be simply measured as “Pearson correlation coefficient”, i.e., $c_{i}=\frac{\langle \left(X_{i}\left(t\right)-\langle X_{i}\left(t\right)\rangle \right)\left(N_{i}\left(t\right)-\langle N_{i}\left(t\right)\rangle \right)\rangle }{\sigma_{X}\sigma_{N}}$, where the angled brackets indicate a time average [24]. However, in certain instances it would be more useful to exploit “Spearman correlation coefficient”, as it more accurately measures how two quantities are predictive of each other.

Averaging the population coupling over many neurons within a large network yields the average population coupling, which represents a measure of the overall level of coordination within the network. The change in mean response was computed as the difference in the mean spike counts over many trials of ongoing activity followed by evoked activity. In our simulations, the ongoing activity is simulated with an arbitrarily chosen external input of *η* = 1/(5*N*), whereas the evoked activity is simulated with *η* = 2/*N*.

## Results

**Fig 1:**
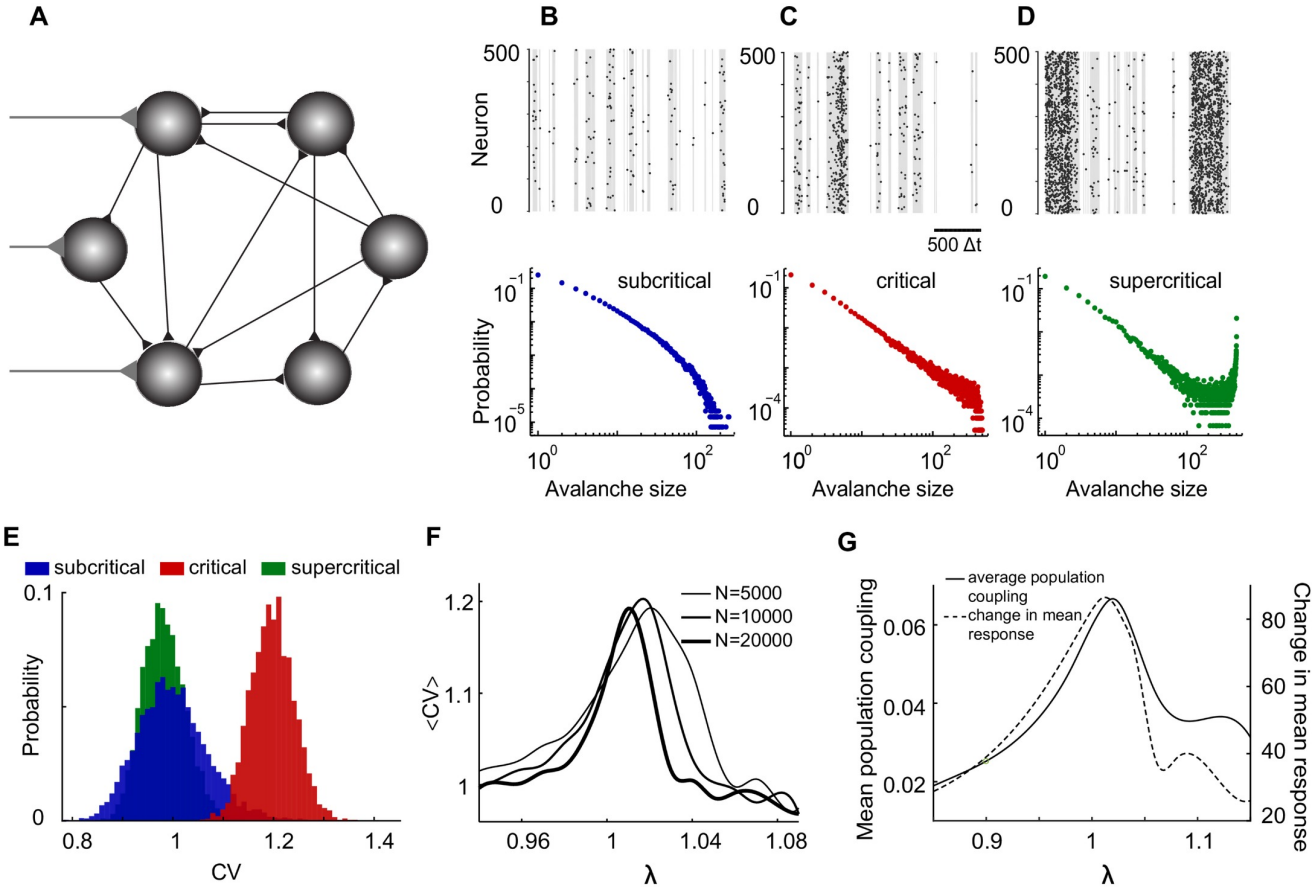
Irregular spiking emerges in a recurrent network operating at criticality. **A**: The model network consists of binary probabilistic model neurons with sparse connectivity (black) and weak external inputs (gray) to a fraction of the neurons. **B-D**: Schematic illustration of avalanche statistics. Simulated spike trains (black raster), neuronal avalanches (gray), and corresponding avalanche size distributions (for 5 × 10^5^ simulation time-steps) for a network of *N* = 500 neurons with 10% connectivity and external inputs *η* = 1/(10*N*) to all neurons. Simulations were performed for three different network states: subcritical ((B) λ = 0.9, blue), critical ((C) λ = 1.0, red), and supercritical ((D) λ = 1.1, green). **E**: Inter-spike-interval CV distributions of simulated spike trains from a network of *N* = 5000 neurons with 3% (arbitrary) connectivity and external inputs *η* = 1/(5*N*) to all neurons. Simulations were conducted for the subcritical (λ = 0.9, blue), critical (λ = 1.02, red), and supercritical (λ = 1.06, green) network states. It should be noted that to better account finite-size effect, the critical point λ = 1.02 is estimated based on the peak for population coupling **F**: The average CV as a function of the maximum eigenvalue λ of the transition probability matrix *P*_*ij*_ for three network sizes. Connectivity was (arbitrarily) chosen to be 3% and external input was 1/(5*N*) to all neurons. The curves were based on 13 values of λ within the range shown and were smoothed using Matlab spline. **G**: The average population coupling and the change in mean response as a function of λ for a network of *N* = 5000 neurons with 3% connectivity and external inputs of strength *η* = 1/(5*N*) applied to all neurons. The use of spline is only to better illustrate the shift of the peaks. To compute the change in mean response, we increased the external input strength by a factor of 10 half-way through the simulation, i.e., from *η* = 1/(5*N*) to *η* = 2/*N*.

To explore the impact of critical dynamics on the statistics of single-neuron spiking, we used a minimal network model consisting of excitatory binary probabilistic neurons with sparse connectivity and external inputs (see Fig 1A and Materials and methods). This model, which is analytically tractable, permits the use of the maximum eigenvalue λ of the *transition probability matrix* *P*_*ij*_ as a control parameter to tune the network at or out of the critical point; λ = 1 indicates the critical point at infinite-size limit, whereas λ < 1 (λ > 1) represents sub-criticality (super-criticality) [22, 23]. As a result, the model is the most natural extension of a branching process to recurrent networks, which is also known to be in the same universality class of directed percolation (see (Fig 1B–1D)).

To investigate the hypothesized impact of the network state on the statistics of neuronal spiking, we simulated the network activity for different values of λ and quantified the single-neuron spiking statistics using mean CV of the inter-spike-interval distributions (Fig 1E). The CV is defined as the ratio of the standard deviation and the mean of the inter-spike-interval distribution for a given neuron. The irregular spiking is basically characterized by *CV* > 1, whereas *CV* = 1 is considered as Poisson spiking. Simulations of large network sizes reveals that near the critical network state (λ ≈ 1) and at the presence of slow drive (see Materials and methods) the CV values are distributed around a mean greater than one, thus indicating irregular spiking.

In contrast, small deviations of the network state towards either the subcritical or the supercritical regime resulted in CV values distributed around a mean of 1 or less. In summary, when tuning the network from the subcritical to the supercritical state, the mean CV peaks near but slightly above λ = 1 (Fig 1F). Moreover, with increasing network size, the peak moves closer towards λ = 1 and becomes narrower as well. This observation suggests that, at the large-size limit, the irregular spiking becomes an emergent property of recurrent networks operating at the critical point (λ = 1).

To further test whether the deviation of the peak from λ = 1 is indeed due to the finite size effect, we looked at two major characteristics of criticality, that are maximum pair-wise correlations and mean response to external stimuli. In order to see how the average correlation among neurons peaks in terms of the control parameter λ, we computed a commonly-used measures of coordinated network activity, namely *average population coupling*. This quantity represents a measure of the overall level of correlated fluctuations within the network [24], which is known to be maximized at criticality (see Materials and methods). Comparing mean CV and average population coupling reveals that they both peak similar to the change in mean response (close to λ = 1; see Fig 1G). This demonstrates that with a finite-sized network, the peak for mean CV coincides with the critical point, therefore, it could be regarded as a hallmark of criticality.

The observed onset of irregular spiking near criticality (Fig 1F) has an intuitive explanation in the extreme limits of connection strength. In the limit of weak connections (λ < 1), due to short lasting nature of neural activity, whenever a cascade of activity is initiated or passed from a single neuron, the chance of recurring spikes becomes negligible, as the activity does not last enough for the same neuron to receive recurrent activity during the same cascade. Therefore single neuron spiking is largely driven by the Poisson external input alone, thus resulting in Poisson like spiking (〈*CV*〉 = 1). On the other hand, neurons become mostly active leading to more regular spiking in the limit of very strong connections (λ > 1), as the network activity becomes saturated; most neurons tend to spike all the time unless it is bounded by their refractory period. But it is at criticality (λ ≈ 1) that the onset of long (power law distributed) cascades of activity leads to more recurrence of spiking during each cascade, thereby resulting in more variability in single neuron spiking. As a result, the scale-free fluctuations in network activity translate to single-neuron irregular spiking (〈*CV*〉 > 1).

Assuming that spike irregularity arises due to the onset of large (sporadic) recurrent activity, one should expect that neurons who receive more input from the network show more variability in their spiking activity. One major factor that determines the amount of network-to-neuron input is a neuron’s in-degree, i.e., the number of input connections to a neuron. Therefore, our conjecture predicts a positive correlation between neurons’ in-degree and their CV.

**Fig 2:**
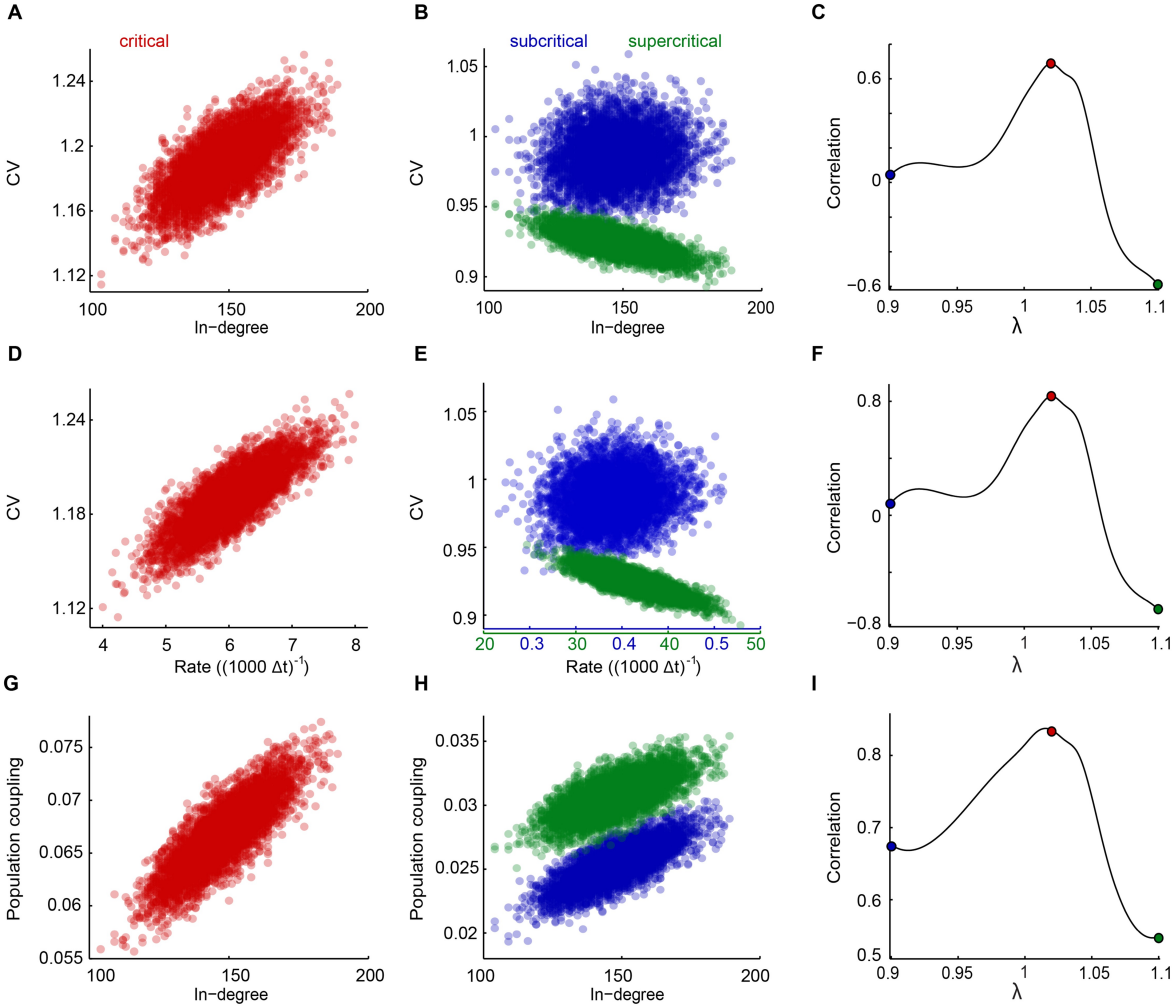
Neuron’s CV’s maximally correlates with their in-degrees and firing rates. **A-B**: The inter-spike-interval CVs from simulated spike trains versus the neuron’s in degree for a model network in three different states: (A) critical (λ = 1.02), (B) subcritical (λ = 0.9) and supercritical (λ = 1.1). Network parameters were *N* = 5000, 3% connectivity, and *η* = 1/(5*N*) applied to all neurons. The approximate critical point (λ = 1.02) is obtained in terms of the peak of population coupling. **C**: Correlation between CV and in-degree as a function of λ. Other network parameters as in (A), (B). The curves were smoothed using Matlab spline to better visualize the peak. The colored dots correspond to the distributions in(A) and (B). **D-E**: *CV* versus rate for three network states. Network parameters as in (A), (B). **F**: Correlation between CV and rate as a function of λ. Other network parameters as in (A), (B). **G-H**: Population coupling versus in-degree for three network states. Network parameters as in (A), (B). **I**: Correlation between population coupling and in-degree as a function of λ. Other network parameters as in (A) and (B).

To test whether such a correlation exists, we took advantage of the distribution of in-degrees provided by a model network and looked into the relation between neuron’s in-degree and their CV. It turned out that even for a random network at criticality (λ ≈ 1), a neuron’s CV tends to increase with increasing in-degree (Fig 2A). Importantly, this correlation between a CV and its connectivity (in-degree) changed drastically when tuning the network state away from criticality. In the subcritical regime only weak correlation was found and in the supercritical regime the relation reversed, i.e., a neuron’s CV tended to decrease with increasing in-degree (Fig 2B). These results could be understood by considering the fact that at the sub-critical regime, the role of external drive becomes more dominant (compared to network recurrent activity), thus the correlation between CV and in-degree vanishes. To the contrary, at the highly super-critical regime neurons with higher in-degree receive more input and their firing rates become more saturated. As a result, they spike more regularly, leading to anti-correlation between neuron’s CV and in-degrees. Accordingly, when tuning the network state through the critical regime, the relation between a neuron’s CV and its in-degree transforms from small correlation in the subcritical regime (λ < 1), to maximized correlation at the critical point (λ ≈ 1), and subsequently to anti-correlation in the supercritical regime (λ > 1), (Fig 2C).

The relationship that solely near criticality is the irregular spiking maximally reflective of the neuron’s in-degree, suggests a novel measure of critical dynamics that could be applicable for a wide range of problems, including neural systems. It is of practical importance that in a network dominated by excitatory neurons, a neuron’s firing rate scales with its in-degree and that this relation is independent of the network state. Thus, spike train recordings from a population of neurons can be informative about the network state: a maximum correlation between a neuron’s CV and its firing rate is indicative of a critical network state, whereas weaker correlation or anti-correlation is indicative of the subcritical or supercritical network state, respectively (Fig 2D and 2F).

Similarly, a neuron’s in-degree also scales with its “population coupling”, thereby resulting in a maximum correlation between CV and “population coupling”. In comparison, the relation between a neuron’s population coupling (see Materials and methods) and its in-degree turns out to be less decisive about the network state, as population coupling increases with a neuron’s in degree for all three network states (Fig 2G and 2H). However, this relation is still prominent for the critical network state (Fig 2I).

**Fig 3:**
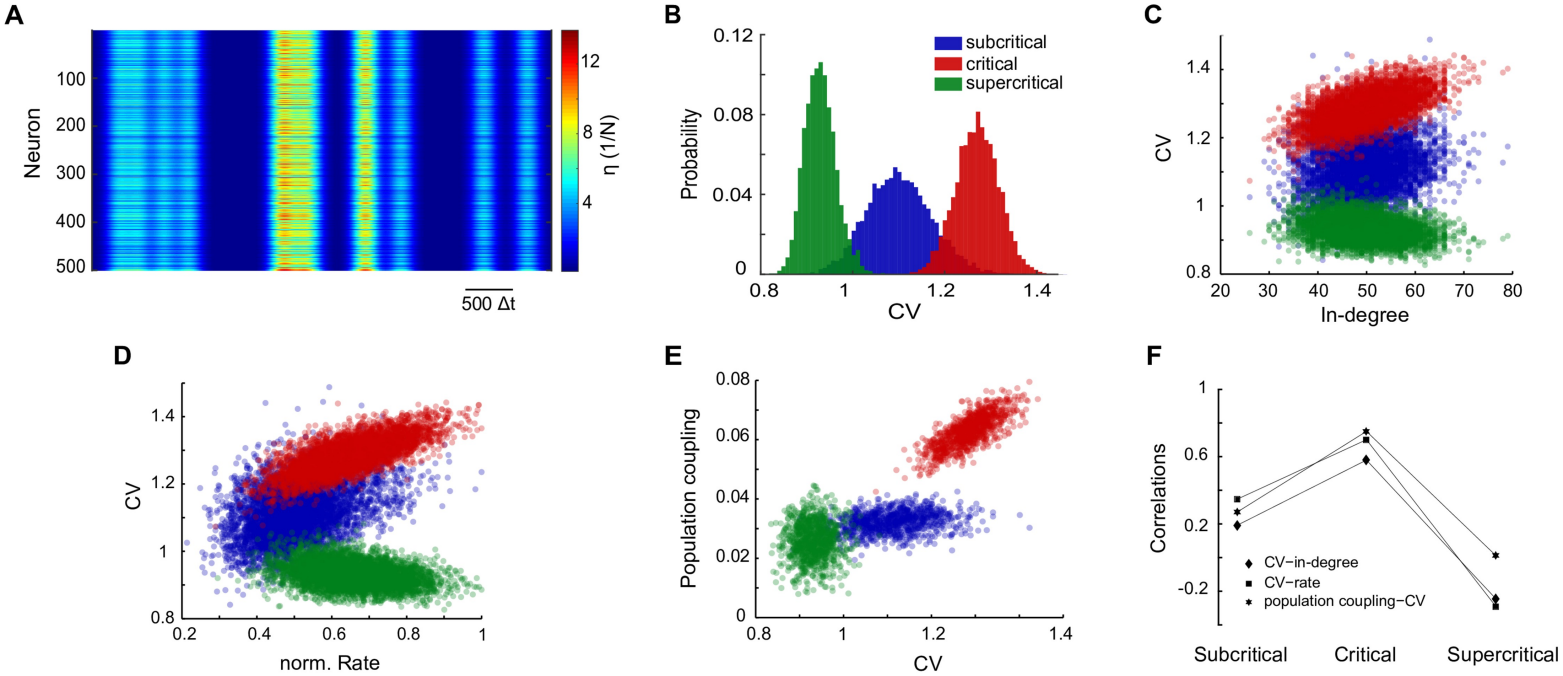
Irregular spiking is maximized at criticality even in the presence of other irregular synchronous drive. **A**: The temporal structure and strength of the external drive *η*(*t*) to 10% of the neurons in a recurrent model network of 5000 neurons and 1% connectivity. The external input *η*(*t*) was generated from Poisson pulses of rate 10/*N*, smoothed by a Gaussian filter of width 100 time-steps and amplitude of 0.2(1 + *ζ*(*t*)), where *ζ*(*t*) is drawn from a normal distribution (see Materials and methods), though the results are not sensitive to the choice of Gaussian filter width or the 0.2 coefficient for *ζ* (see panel D in S4 Fig). This synchronous input was added to a background constant external input of 1/(10*N*). **B**: Inter-spike-interval CV distributions of simulated spike trains for the subcritical (λ = 0.95, blue), critical (λ = 1.02, red), and supercritical (λ = 1.09, green) network state. Although even at the subcritical state the network shows irregular spiking indicated by a mean CV significantly larger than 1, the spike trains show highest irregularity at the critical regime, which is indicated by the peak of the CV distribution located near 1.3. **C-D**: The inter-spike-interval CVs from simulated spike trains versus the neuron’s in degree (C) and its normalized firing rate (D) for the three network states. **E**: The population coupling vs a neuron’s CV for the three network states. **F**: The Spearman correlation between different pairs of single neuron measures, indicating that neurons’ CV maximally correlate with their in-degree, firing rate and population coupling.

We have shown so far that irregular spiking could be an emergent property of a critical recurrent neural network at the presence of constant (stochastic) external drive. In addition, we have shown that criticality maximizes the correlation between the neuron’s in-degree and CV. However, the above results could be confounded by the fact that there are numerous possible mechanisms for generating spike irregularity. Of the most trivial mechanisms is the existence of some sort of double stochasticity, such as the presence of a synchronous inhomogeneous Poisson drive (see Discussion and Fig 3), by which one can easily generate irregular spiking (*CV* > 1) even in a sub-critical network (see Fig 3). Regarding such possibilities, we show that the impact of the network state on the statistics of single-neuron spiking is robust to the nature of the external drive (Fig 3, S1, S2, S3 and S4 Figs). Furthermore, we also show that our results are largely robust to several parameters such as the mean network connectivity and refractory period (S4 and S5 Figs).

## Discussion

The investigation of criticality in biological networks has a long history and continues to flourish [25–32]. In particular, a wealth of evidence suggests the hypothesis that neural networks of cerebral cortex operate at criticality [2, 3, 5, 7, 9, 33]. This hypothesis, is largely supported by the observation of power law distributed avalanche sizes and durations with respective exponents of 1.5 and 2.0. The existence of such critical exponents has led to the idea that the spread of activity in biological neural networks could be well described by branching processes, thereby making them belong to the universality class of directed percolation. Theoretical studies have also lent credence to this idea, as it is shown that the general dynamical equations describing coarse-grained neural activity could exhibit a DP phase transition [17]. In addition, it has been shown that being at a critical regime brings about several advantages for information processing [18, 19, 21, 34, 35]. However, despite its plausibility, the neural criticality remains to be controversial due to multiple reasons: First, most of its experimental evidence has been based on power law scaling of avalanche statistics, which is not sufficient for inferring criticality. This issue becomes even more serious as it is known that in addition to the critical exponents for size and duration of avalanches, the same scaling relation between them could exist even in systems that show no critical phase transition [36]. Secondly, although avalanche statistics may serve as a statistical signature of critical dynamics, to what degree it predicts certain universal features of cortical activity has yet to be understood. This demonstrates the need for having a more in-depth study of the relation between criticality and other elements of neural activity, as well as having some complementary measures of criticality. Additionally, performing conventional avalanche analysis usually entails some preprocessing and ad hoc procedures such as applying an optimal time bin of network inter-event interval [2], for which there has been no solid theoretical underpinning. This has been another source of controversy and raises the need for some complementary hallmarks of criticality which could be measured more directly from neural data.

Here, using a minimal model in the DP universality class, we showed that irregular spiking and its relation to the neuron’s in-degree could result as emergent properties of a recurrent network operating at criticality. Our results not only provide an insight on some implications of critical dynamics, but they also suggest new hallmarks of criticality in the general context of excitatory recurrent networks. Furthermore, these results could be of particular importance in neuroscience: assuming that at coarse-grained scale cortical networks belong to DP universality class, our results would relate two universal features of cortical activity; the scale invariant dynamics at the population level and spike irregularity at the single-neuron level.

A number of separate dynamical, biophysical, and structural mechanisms have been proposed to generate the observed irregular spiking in neural data [37–40]. It has long been known that a leaky integrate and fire (LIF) neuron residing at the sub-threshold regime can exhibit Poisson-like spiking *CV* ≈ 1, during which the membrane potential is just below the threshold and the spiking activity is mostly driven by current fluctuations [15]. Recently, it has been also shown that depending on the shape of the single-neuron transfer function and synaptic time scales, balanced networks of leaky integrate and fire LIF neurons are capable of showing a continuous phase transition [16]. For such transition, it turns out that the mean *CV* acts as an order parameter, as the network undergoes a transition from a phase of Poisson spiking to irregular spiking (*CV* > 1) when the average synaptic efficacy crosses a critical value [16]. Therefore, in these models the onset of irregular spiking could be easily achieved for a large range of parameters. However, whether and under what circumstance these models can show scale invariant activity, as observed in the cortex, is still unknown.

The significance of our work resides in part in proposing an alternative scenario, in which irregular spiking simply emerges from the collective behavior of excitatory neurons, without the need for exploiting any further detailed mechanism at the single neuron level. Indeed, experimental evidence for the predicted coexistence of irregular spiking and criticality (in the DP sense) has been already provided in recordings of ongoing cortical activity in vivo [10, 41], but a universal mechanism underlying such coexistence has been missing. Also, while the coexistence of irregular spiking and power-law avalanche size distributions has been demonstrated in more complex model networks [42–45], our work extends qualitatively beyond these important earlier studies in certain fundamental aspects. First, the choice of a network of excitatory probabilistic integrate-and-fire model neurons allows the precise analytic evaluation of the network state via a single control parameter λ, i.e., the maximum eigenvalue of the transfer probability matrix. This approach avoids the need to rely on the cumbersome and less precise measures such as avalanche analysis or “branching ratio” (see [2] to evaluate the network state). Second, we show that spike irregularity and power law avalanches not only coexist, but they could be two features of the same phenomenon, that is critical dynamics. Finally, our finding that spike irregularity maximally correlates with firing rate provides an important new measure of criticality. Consistent with these model results, recent experimental data from in vivo recordings showed increased population coupling with increasing synaptic inputs [24]. However, unless the network state is manipulated and a maximum in the correlation between the population coupling and the synaptic input is determined, such experimental fact won’t be informative about the network state.

Despite the applicability of our findings to the general context of network dynamics, there would be certain limitations when it comes to the presence of inhibition. This goes back to the fact that contrary to the DP universality class which has a single control parameter (mean synaptic efficacy in this context), having inhibition adds another key parameter, namely the excitation to inhibition (E/I) ratio. This makes the network dynamics more complicated as the system can behave very differently depending on varying each parameter. Importantly, it is known that depending on the choice of model parameters, LIF neurons can in general shift to a phase of synchronized irregular spiking (SI) by distorting the E/I balance [20]. In addition, it has been shown that performing network disinhibition using perturbation techniques such as pharmacological manipulations could create more synchronized activity, which could conflict with our prediction for a DP phase transition [18, 35]. On the other hand, having a constant E/I ratio leaves open the possibility of a DP transition in which the synaptic efficacy acts as a control parameter. Even so, such possibility leads to a secondary challenge of experimental verifiability, since contrary to E/I balance, manipulating mean synaptic efficacies would be a great experimental challenge. Therefore, the issue of relevance to more realistic neurons, in some sense, boils down to the very question of whether realistic spiking networks are capable of having a DP phase transition. In that view, whether and to what degree our results could be applicable to realistic neural networks remains to be an open question that could be the topic of future studies.

Given the limitations of our model, there are a few points to be mentioned about the merits and downsides of the model. (i) As stated before, the model is the most natural extension of branching processes to recurrent networks, which is also at the same time analytically tractable with a single control parameter. Therefore, given that biological neural networks fit to DP universality class at the coarse-grained level [17], our predictions (on maximization of spike irregularity and its relation to neuronal firing rates) provide us with new measures to verify such hypothesis. (ii) Our results demonstrate that maximum spike irregularity and its correlation with firing rate are emergent properties of recurrent neural networks at the population level. This conclusion could not be easily drawn with a biologically plausible model with many free parameters. (iii) Although the model might sound too simple to account for real neural systems, under general conditions even real data from excitatory neurons could be mapped to such a model, as the transfer probabilities can be inferred using either transfer entropy or Granger causality (for example see [46]). However, it should be noted that the use of such data driven modeling is contingent on the assumption of tree-like propagation of activity, which has its own limitations (see Methods). (iv) Our results go beyond the realm of neuroscience, as they should be applicable to any network of excitatory units.

To avoid some common misconceptions, it is worth to point out that contrary to this study, in the neuroscience community, the phrase “irregular spiking” is commonly used in a loose sense. Mathematically, *CV* = 1 is a signature of Poisson spiking, by which the distribution of inter-spike-interval becomes exponential. To the contrary, irregular (regular) spiking is referred to the case where *CV* > 1 (*CV* < 1). That being said, here we refer to “irregularity” in the strict sense as each three cases of *CV* > 1, *CV* = 1 and *CV* < 1 indicate spike trains with fundamentally different characteristics. Although the evidence from cortical activity in vivo is consistent with *CV* > 1, it is common for many computational models to fail to capture such distinction between irregular vs Poisson spiking. We believe it is essential to avoid such a loose language and demand any model of cortical activity to exactly mimic the statistics of real spike trains, rather than putting any statistics with *CV* ≈ 1 into the same category of irregular spiking.

One important future step to build on our study is to see whether the observation that spike irregularity is maximized at criticality could be derived analytically. Although, we show that our results are robust to several factors such as the type of the external input, refractoriness, and connectivity (see S4 and S5 Figs), there is no doubt that having analytical results would substantiate our results more rigorously and sheds light on the exact relation between the network state and single-neuron spiking.

In conclusion, our study proposes complementary measures of criticality that have certain advantages to other common measures such as power law scaling, as they are more robust to subsampling, and are also easy to measure directly from neural data. Moreover, depending on the validity of the criticality hypothesis, they provide us with an insight on the impact of critical neural dynamics at the single-neuron level. We believe future studies on possibility of (DP) critical phase transitions in realistic spike models will shed more light on this matter.

## Supporting information

S1 Fig The relation between population coupling and firing rate in the presence of synchronous external drive. **A**: The temporal structure and strength of the external input *η*(*t*) to 10% of the neurons in a recurrent model network of 5000 neurons and 1% connectivity. The external input *η*(*t*) was generated from Poisson pulses of rate 10/*N*, smoothed by a Gaussian filter of width 100 time-steps and amplitude of 0.2(1 + *ζ*_*t*_), where *ζ*_*t*_ is drawn from a normal distribution (see Materials and methods). This synchronous input was added to a background constant external input of 1/(10*N*). **B**: Inter-spike-interval CV distributions of simulated spike trains for the subcritical (λ = 0.95, blue), critical (λ = 1.02, red), and supercritical (λ = 1.09, green) network state. At the critical regime the spike trains show highest irregularity, which is indicated by the peak of the CV distribution located near 1.3. **C-D**: The population coupling from simulated spike trains versus the neuron’s in degree (C) and its normalized rate (D) for the three network states. **E**: The population coupling vs a neuron’s CV for the three network states. **F**: The Spearman correlation coefficients between CV and in-degree (rate), population coupling and in-degree (rate), population coupling and CV are all maximized at criticality.
(TIF)

S2 Fig Irregular spiking at criticality in a recurrent network with asynchronous external input. **A**: The temporal structure and strength of the external input *η*(*t*) to 10% of the neurons in a recurrent model network of 5000 neurons and 1% connectivity. The external input *η*(*t*) was generated by independent Poisson pulses of rate 5/*N*, smoothed by a Gaussian filter of width 20 time-steps and amplitude *η*_0_ = 0.5 (see Materials and methods). **B**: Inter-spike-interval CV distributions of simulated spike trains for the subcritical (λ = 0.95, blue), critical (λ = 1.02, red), and supercritical (λ = 1.09, green) network state. **C-D**: The inter-spike-interval CVs from simulated spike trains versus the neuron’s in degree (C) and its normalized rate (D) for the three network states. **E-F**: The population coupling from simulated spike trains versus the neuron’s in degree (e) and its normalized rate (f) for the three network states. **G**: The population coupling vs a neuron’s CV for the three network states. **H**: The Spearman correlation coefficients between CV and in-degree (rate), population coupling and in-degree (rate), population coupling and CV are all maximized at criticality.
(TIF)

S3 Fig The mean CV and average population coupling are maximized near network criticality for external inputs of different spatiotemporal structure. **A-B**: Average CV (A) and average population coupling (B) vs the control parameter λ for synchronous external inputs (see S1A Fig, but with different stimulation amplitudes (see panel A in S1 Fig, but with stimulation amplitude *η*_0_ = 0.1, 0.2, 0.5; see Materials and methods) for three different network sizes. **C-D** Average CV (C) and average population coupling (D) vs the control parameter λ for synchronous external inputs (see panel A in S1 Fig) for a network size of *N* = 5000 and for three different stimulus amplitudes. **E-F** Average CV (E) and average population coupling (F) vs the control parameter λ for random (asynchronous) external input (see panel A in S2 Fig) for three different network sizes (see legend in (A)).
(TIF)

S4 Fig Robustness of maximum irregularity to model parameters. **A**: CV profiles for different refractory periods in networks of 5000 neurons (*η* = 1/5*N*). The mean CV clearly shows a pronounced peak for a range of refractory periods, though with very large refractoriness (*τ*_*ref*_ ≈ 10) the profile becomes almost flat. **B**: CV profiles vs constant external drive for networks of 5000 neurons with %1 connectivity and *τ*_*ref*_ = 2. It is evident that a separation of time scales is necessary to have maximum CV at criticality, as increasing *η* degrades the maximum CV and at some point breaks down irregular spiking (*CV* > 1). **C**: CV profiles vs the parameters of asynchronous drive (*η*_0_ and the width of the Gaussian filter; see also Materials and methods). Similar to the case of constant drive, spike irregularity is degraded by increasing *η*_0_, though it largely persists (*CV* > 1) for a range of *η*’s. On top of that, the CV profiles are highly robust to the choice of Gaussian filter. **D**: CV profiles vs the parameters of synchronous drive (*ϵ* and the width of the Gaussian filter; see also Materials and methods). The spike irregularity turns out to be insensitive to both parameters.
(TIF)

S5 Fig Robustness of general results to network connectivity. **A**: CV profiles for different connectivities (mean degrees) for random networks of 5000 neurons with external input of *η* = 1/5*N*. It is evident that the results are highly robust to connectivity. **B**: Similar to A for the average population coupling (PC).
(TIF)
