# Supplementary figures and images for: Criticality predicts maximum irregularity in recurrent networks of excitatory nodes
Source: PLoS One. 2017 Aug 17;12(8):e0182501. doi: 10.1371/journal.pone.0182501 (PMC5560579; doi:10.1371/journal.pone.0182501)

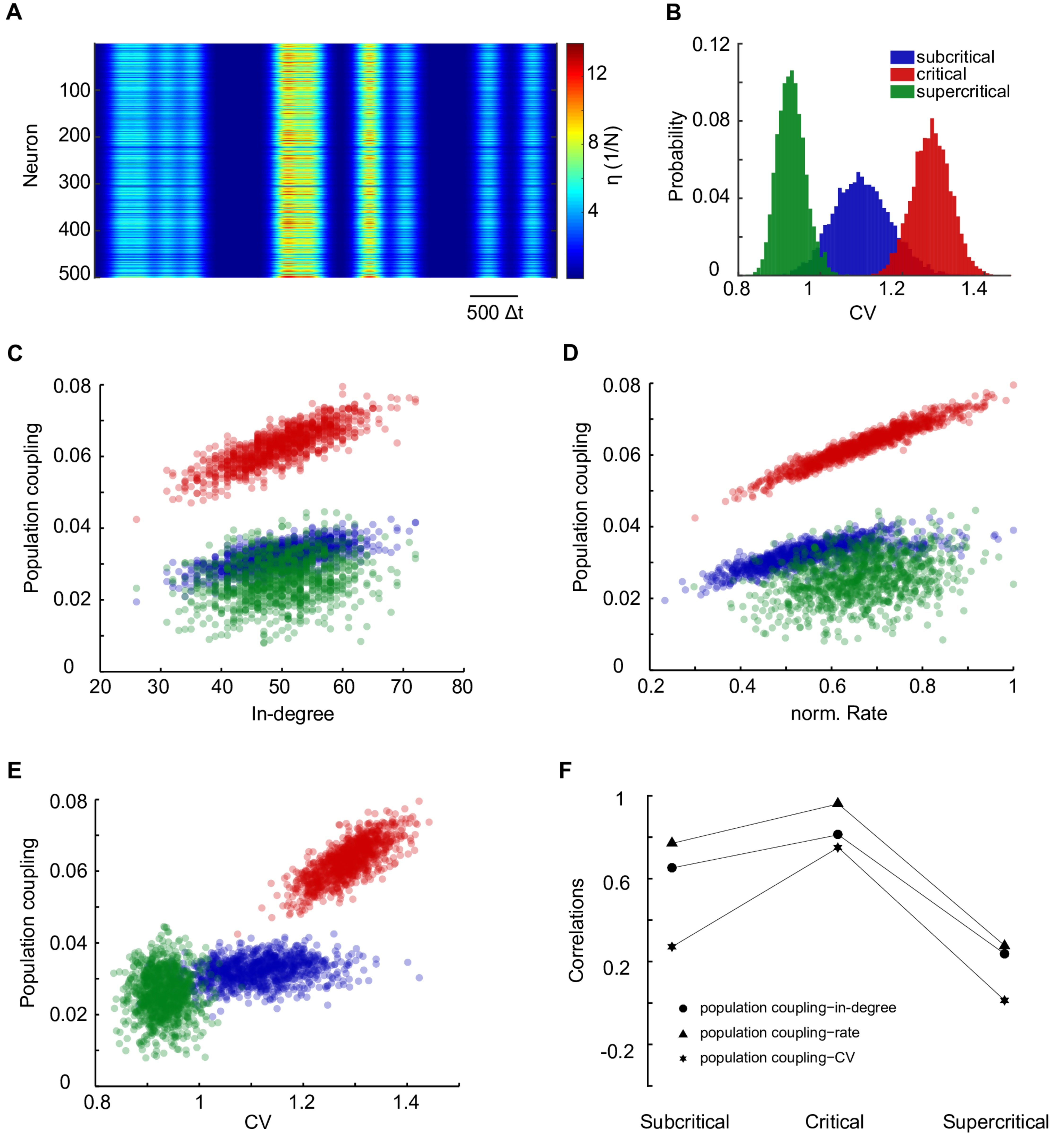

Supplement: S1 Fig — A: The temporal structure and strength of the external input η(t) to 10% of the neurons in a recurrent model network of 5000 neurons and 1% connectivity. The external input η(t) was generated from Poisson pulses of rate 10/N, smoothed by a Gaussian filter of width 100 time-steps and amplitude of 0.2(1 + ζt), where ζt is drawn from a normal distribution (see Materials and methods). This synchronous input was added to a background constant external input of 1/(10N). B: Inter-spike-interval CV distributions of simulated spike trains for the subcritical (λ = 0.95, blue), critical (λ = 1.02, red), and supercritical (λ = 1.09, green) network state. At the critical regime the spike trains show highest irregularity, which is indicated by the peak of the CV distribution located near 1.3. C-D: The population coupling from simulated spike trains versus the neuron’s in degree (C) and its normalized rate (D) for the three network states. E: The population coupling vs a neuron’s CV for the three network states. F: The Spearman correlation coefficients between CV and in-degree (rate), population coupling and in-degree (rate), population coupling and CV are all maximized at criticality. (TIF) [file pone.0182501.s001.tif]

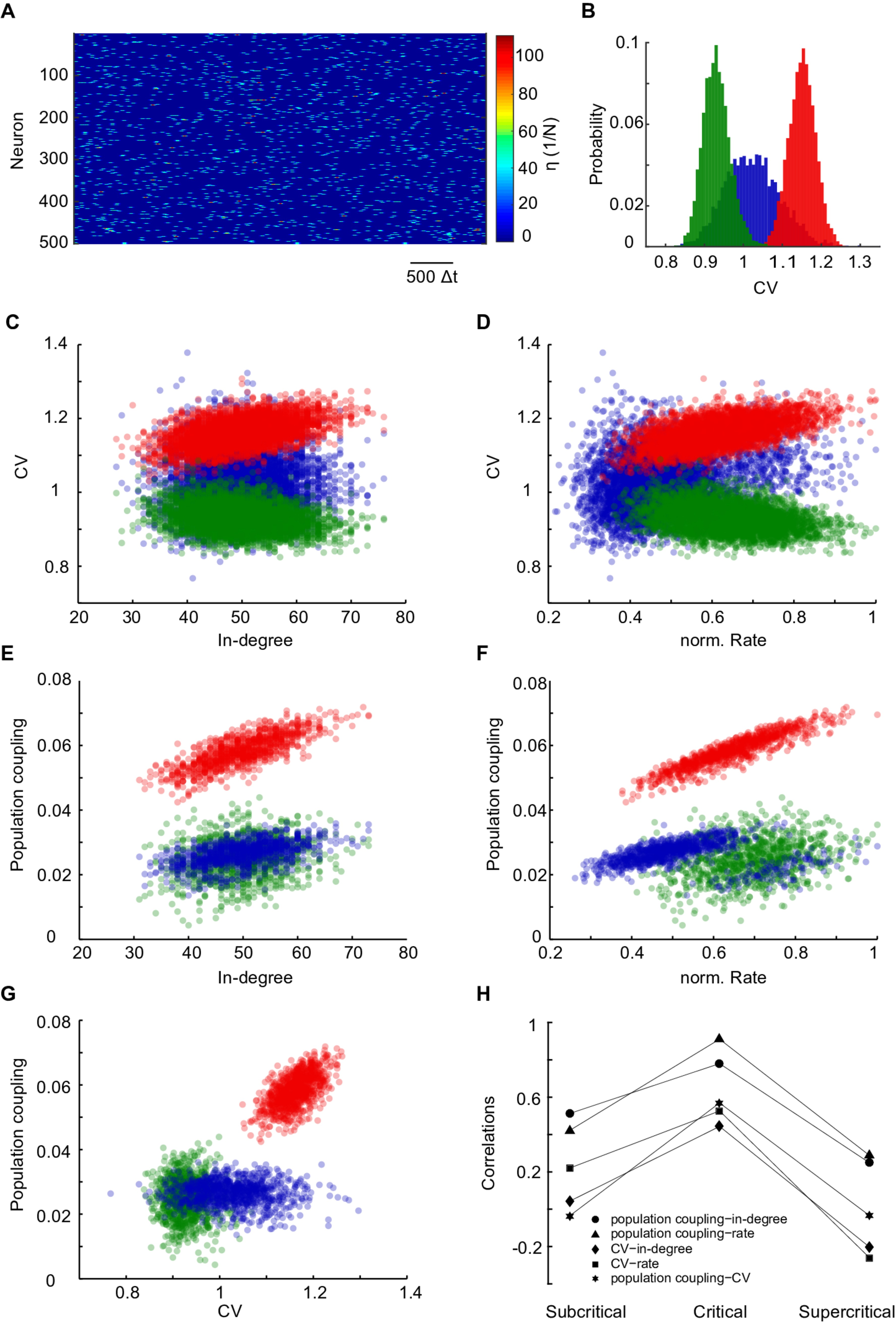

Supplement: S2 Fig — A: The temporal structure and strength of the external input η(t) to 10% of the neurons in a recurrent model network of 5000 neurons and 1% connectivity. The external input η(t) was generated by independent Poisson pulses of rate 5/N, smoothed by a Gaussian filter of width 20 time-steps and amplitude η0 = 0.5 (see Materials and methods). B: Inter-spike-interval CV distributions of simulated spike trains for the subcritical (λ = 0.95, blue), critical (λ = 1.02, red), and supercritical (λ = 1.09, green) network state. C-D: The inter-spike-interval CVs from simulated spike trains versus the neuron’s in degree (C) and its normalized rate (D) for the three network states. E-F: The population coupling from simulated spike trains versus the neuron’s in degree (e) and its normalized rate (f) for the three network states. G: The population coupling vs a neuron’s CV for the three network states. H: The Spearman correlation coefficients between CV and in-degree (rate), population coupling and in-degree (rate), population coupling and CV are all maximized at criticality. (TIF) [file pone.0182501.s002.tif]

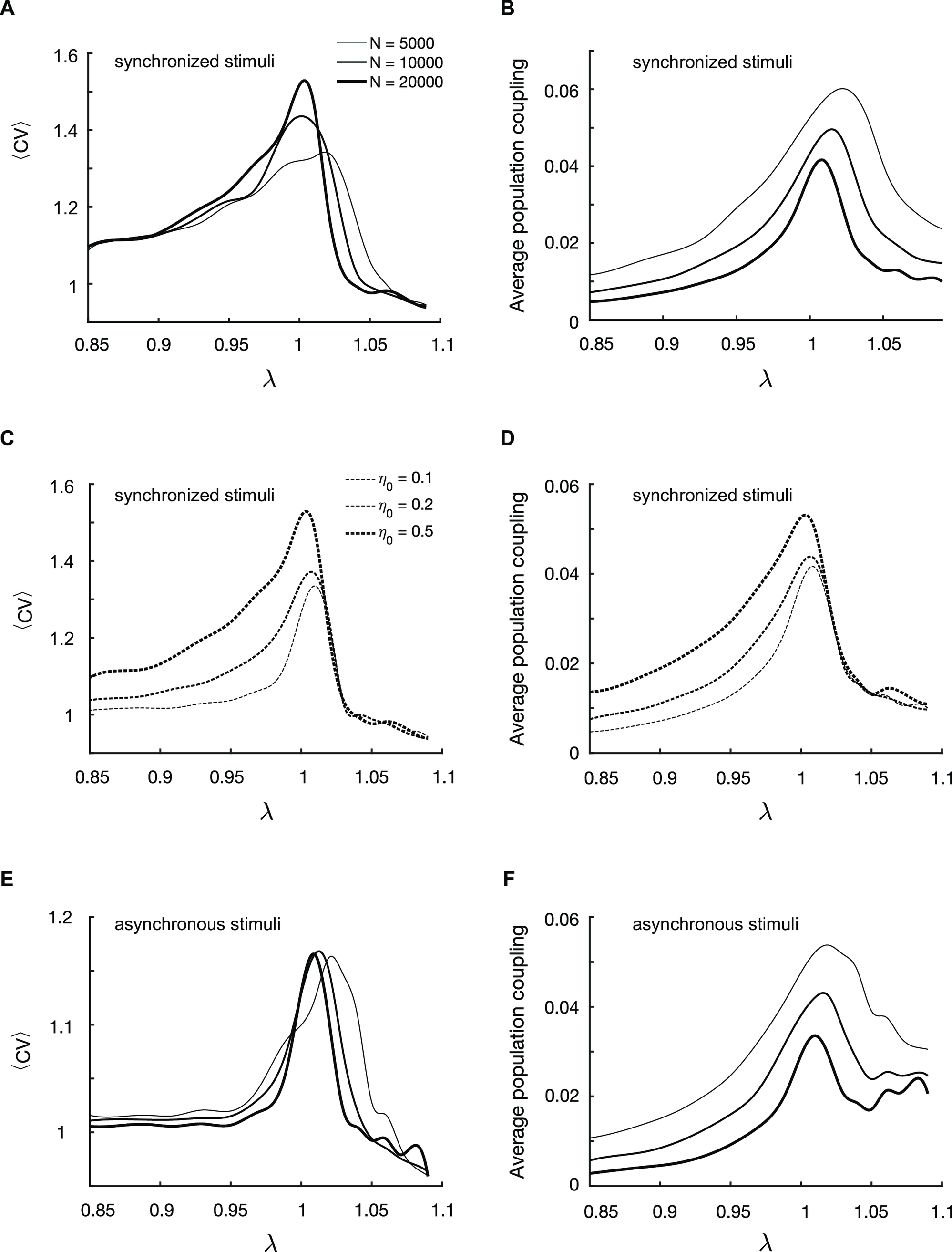

Supplement: S3 Fig — A-B: Average CV (A) and average population coupling (B) vs the control parameter λ for synchronous external inputs (see S1A Fig, but with different stimulation amplitudes (see panel A in S1 Fig, but with stimulation amplitude η0 = 0.1, 0.2, 0.5; see Materials and methods) for three different network sizes. C-D Average CV (C) and average population coupling (D) vs the control parameter λ for synchronous external inputs (see panel A in S1 Fig) for a network size of N = 5000 and for three different stimulus amplitudes. E-F Average CV (E) and average population coupling (F) vs the control parameter λ for random (asynchronous) external input (see panel A in S2 Fig) for three different network sizes (see legend in (A)). (TIF) [file pone.0182501.s003.tif]

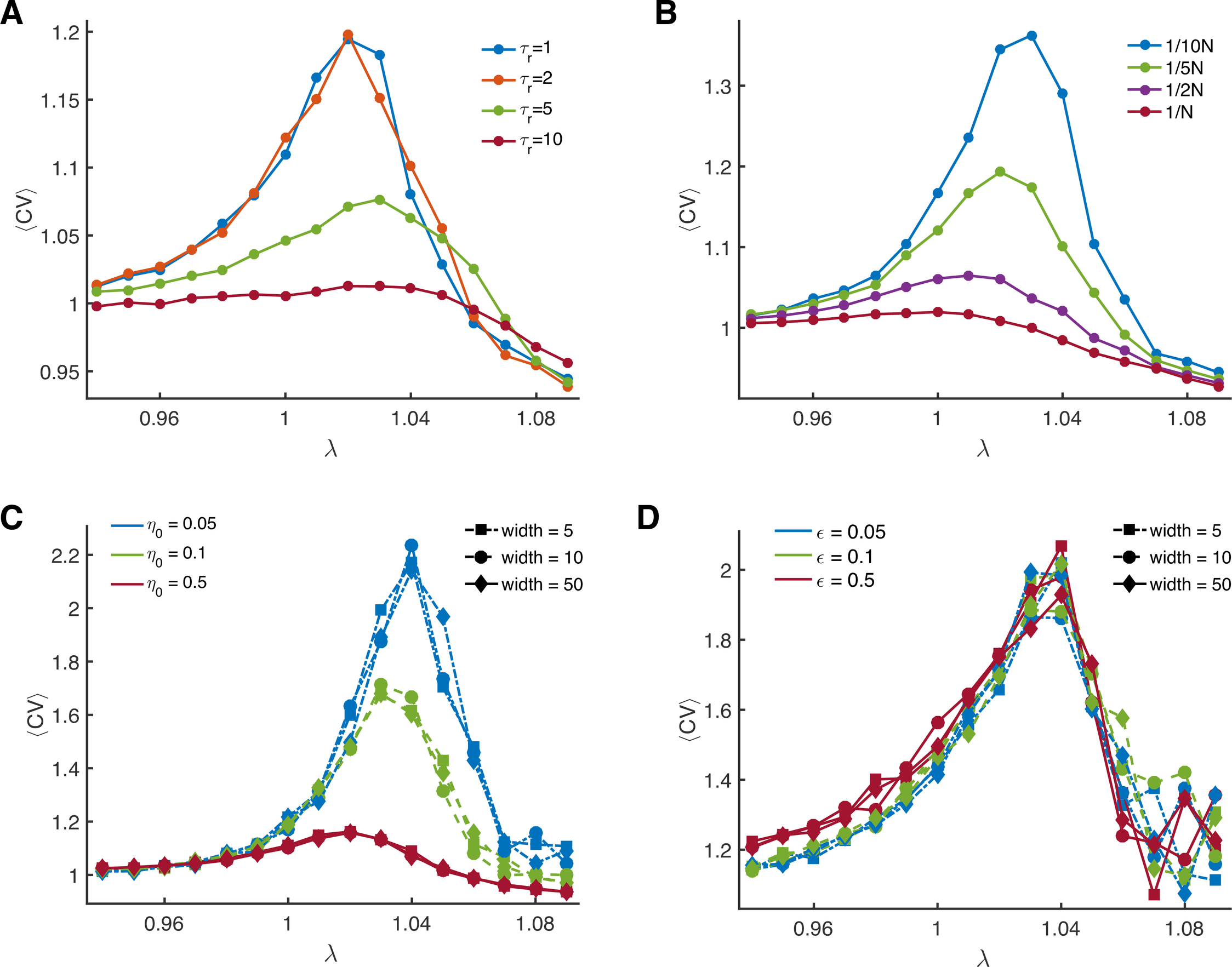

Supplement: S4 Fig — A: CV profiles for different refractory periods in networks of 5000 neurons (η = 1/5N). The mean CV clearly shows a pronounced peak for a range of refractory periods, though with very large refractoriness (τref ≈ 10) the profile becomes almost flat. B: CV profiles vs constant external drive for networks of 5000 neurons with %1 connectivity and τref = 2. It is evident that a separation of time scales is necessary to have maximum CV at criticality, as increasing η degrades the maximum CV and at some point breaks down irregular spiking (CV > 1). C: CV profiles vs the parameters of asynchronous drive (η0 and the width of the Gaussian filter; see also Materials and methods). Similar to the case of constant drive, spike irregularity is degraded by increasing η0, though it largely persists (CV > 1) for a range of η’s. On top of that, the CV profiles are highly robust to the choice of Gaussian filter. D: CV profiles vs the parameters of synchronous drive (ϵ and the width of the Gaussian filter; see also Materials and methods). The spike irregularity turns out to be insensitive to both parameters. (TIF) [file pone.0182501.s004.tif]

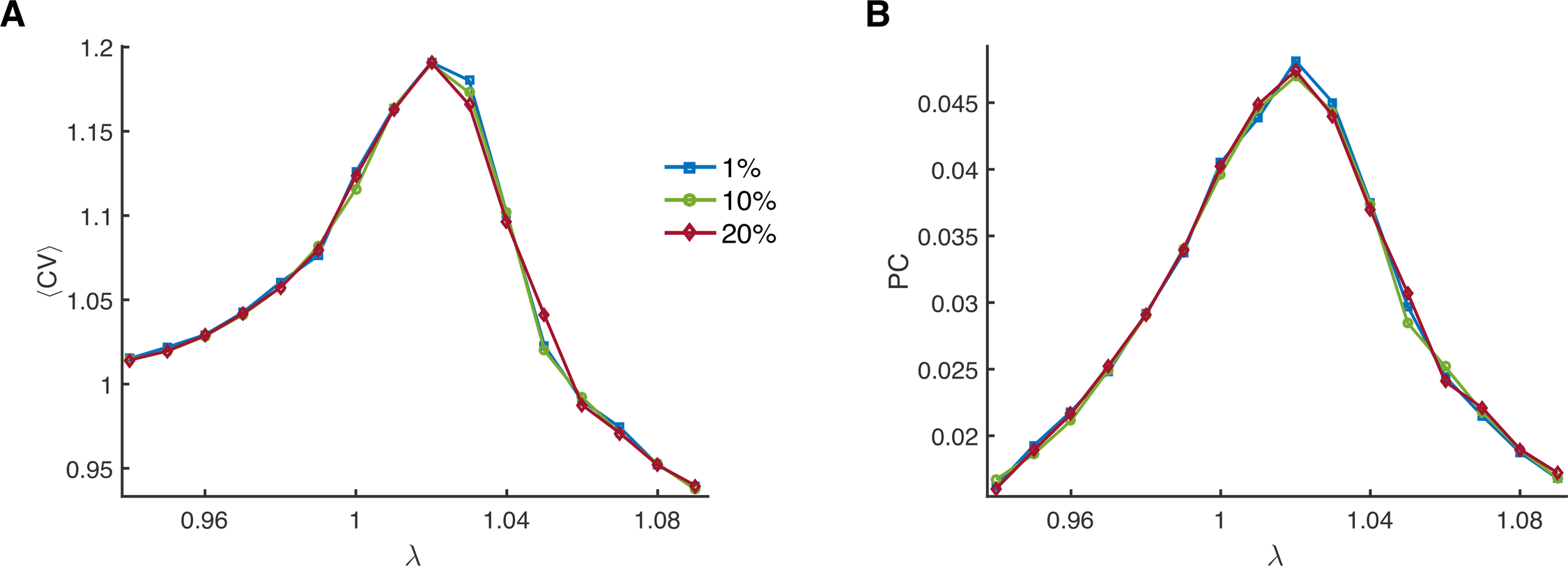

Supplement: S5 Fig — A: CV profiles for different connectivities (mean degrees) for random networks of 5000 neurons with external input of η = 1/5N. It is evident that the results are highly robust to connectivity. B: Similar to A for the average population coupling (PC). (TIF) [file pone.0182501.s005.tif]
